# Chronic rhinosinusitis with nasal polyps: changing expectations

**DOI:** 10.1016/j.bjorl.2024.101415

**Published:** 2024-02-29

**Authors:** Otavio B. Piltcher

**Affiliations:** Depto de Oftalmologia e Otorrinolaringologia da UFRGS, Hamburgh Fellow Univseristy of Pittsburgh School of Medicine, Avenida Bento Gonçalves 8083/2, Porto Alegre, RS, Brazil

Who would have thought 15 years ago that we would discuss the possibility of achieving remission or even a cure for CRSwNP? Who would have thought that the feeling of helplessness common among otorhinolaryngologists when faced with a patient with nasal congestion, anosmia/hyposmia, associated with polyposis, even worse if accompanied by asthma and exacerbated by aspirin, would be replaced by a more hopeful outlook, with much more ambitious expectations?

It is clear that the emergence of biologics, with their direct action in controlling different stages of type 2 inflammation, which is characteristic of these patients, is directly related to this paradigm shift.[1], [2] However, those who believe this is such a simple and straightforward response should be corrected. Currently, the clinical trials that have demonstrated the effectiveness of these drugs live with the sentence of chronic use of a high-cost medication and, as time goes by, of proving the absolute long-term safety of this intervention in the immune system.

It's interesting to note that the emergence of biologics takes place at one of the most successful stages in the history of clinical-surgical treatment for these patients, especially in the face of a combination of complete ethmoidectomy and exposure to more effective washes with topical corticosteroids, even albeit off-label. Is this surgical success true for all cases? The answer is no. Neither, given the current capacity for endotype differentiation in patients, is it possible to guarantee success using biologics in more than 70%–80% of cases, according to the available data.[1], [4]

There is plenty of room for scientific evolution without definitive statements or blind feelings defending one treatment over another. This discussion could become more fruitful as more markers become available daily, enabling the identification of endotypes that respond to different therapeutic options or combinations of them and possible and probable dosages that differ from those authorized and scientifically tested to date. For example, would it be possible to establish new options, such as using preoperative biologics, and then reduce the frequency of use as necessary post-surgical intervention? How effective would these new treatment modalities be? And would the adverse effects of manipulating and damming specific inflammatory lineages for different lengths of time be less than those studied so far?

Although there is justifiable resistance and concern about financial aspects due to the current high cost of these medications, mature decisions will only be viable in the face of cost-effectiveness studies.^3^ Evaluations where surgery as a comparison modality should at least expose the limits of the ethmoid, biologics could be used in different dosage proposals, and the costs associated with the long-term adverse effects of abused oral corticosteroids among these patients should also be weighed up. All this is within the irreversible principle of so-called precision medicine.

In short, this avalanche of news and findings has justified raising the bar regarding the expectations of specialists and their patients. New times, more knowledge, new therapeutic alternatives, new feelings — we are indeed experiencing a new era about CRSwNP.
